# Genetic human prion disease modelled in PrP transgenic *Drosophila*

**DOI:** 10.1042/BCJ20170462

**Published:** 2017-09-20

**Authors:** Alana M. Thackray, Alzbeta Cardova, Hanna Wolf, Lydia Pradl, Ina Vorberg, Walker S. Jackson, Raymond Bujdoso

**Affiliations:** 1 Department of Veterinary Medicine, University of Cambridge, Madingley Road, Cambridge CB3 OES, U.K.; 2 German Center for Neurodegenerative Diseases (DZNE e.V.), Bonn, Germany; 3 Rheinische Friedrich-Wilhelms-Universität, Bonn, Germany

**Keywords:** *Drosophila melanogaster*, genetic, neurotoxicity, prion, PrP^Sc^, transmissible

## Abstract

Inherited human prion diseases, such as fatal familial insomnia (FFI) and familial Creutzfeldt–Jakob disease (fCJD), are associated with autosomal dominant mutations in the human prion protein gene *PRNP* and accumulation of PrP^Sc^, an abnormal isomer of the normal host protein PrP^C^, in the brain of affected individuals. PrP^Sc^ is the principal component of the transmissible neurotoxic prion agent. It is important to identify molecular pathways and cellular processes that regulate prion formation and prion-induced neurotoxicity. This will allow identification of possible therapeutic interventions for individuals with, or at risk from, genetic human prion disease. Increasingly, *Drosophila* has been used to model human neurodegenerative disease. An important unanswered question is whether genetic prion disease with concomitant spontaneous prion formation can be modelled in *Drosophila*. We have used pUAST/PhiC31-mediated site-directed mutagenesis to generate *Drosophila* transgenic for murine or hamster PrP (prion protein) that carry single-codon mutations associated with genetic human prion disease. Mouse or hamster PrP harbouring an FFI (D178N) or fCJD (E200K) mutation showed mild Proteinase K resistance when expressed in *Drosophila*. Adult *Drosophila* transgenic for FFI or fCJD variants of mouse or hamster PrP displayed a spontaneous decline in locomotor ability that increased in severity as the flies aged. Significantly, this mutant PrP-mediated neurotoxic fly phenotype was transferable to recipient *Drosophila* that expressed the wild-type form of the transgene. Collectively, our novel data are indicative of the spontaneous formation of a PrP-dependent neurotoxic phenotype in FFI- or CJD-PrP transgenic *Drosophila* and show that inherited human prion disease can be modelled in this invertebrate host.

## Introduction

Human prion diseases are a group of fatal transmissible neurodegenerative conditions that may be genetic, acquired or arise sporadically. Collectively, these conditions include Creutzfeldt–Jakob disease (CJD), fatal familial insomnia (FFI), Gerstmann–Sträussler–Scheinker disease (GSS), variant CJD and kuru [1]. Prion diseases are characterised by the conversion of host PrP, the prion protein, from a normal isomer PrP**^C^** into a misfolded and aggregated abnormal conformer PrP**^Sc^**, which accumulates in the brain of affected individuals [2]. Human prion diseases are transmissible, both within the species and to experimental hosts [3]. According to the prion hypothesis, PrP**^Sc^** is the major, if not sole, component of the transmissible prion moiety [4]. Furthermore, expression of PrP**^C^** and its conversion into PrP**^Sc^** are considered necessary for prion-induced neurodegeneration [5–8].

Genetic human prion diseases, such as FFI, familial CJD (fCJD) and GSS, are associated with autosomal dominant mutations in the human prion protein gene *PRNP* [9–13]. More than 30 different pathogenic mutations in *PRNP* have been identified which give rise to the following changes in PrP**^C^**: single amino acid substitution; premature polypeptide stop codon or insertion of extra octapeptide repeats [14]. How these mutations in *PRNP* induce prion disease remains unclear although a generally held view is that they increase the tendency of PrP**^C^** to form PrP**^Sc^** by influencing prion protein structure [15–20]. In this context, mutations in *PRNP* may promote PrP**^C^** misfolding, enhance misfolded PrP to aggregate or increase the stability of PrP**^Sc^**. It is important to identify the molecular pathways and cellular processes that regulate prion formation and prion-induced neurotoxicity. This will allow identification of possible therapeutic interventions for those individuals with genetic human prion disease, or those at risk in the case of asymptomatic carriers of these conditions.

Genetic forms of human prion diseases are difficult to study in the natural host. These conditions are relatively rare and are characterised by a long asymptomatic phase prior to the onset of clinical disease [3]. As a consequence, attempts have been made to model genetic human prion diseases in mice transgenic for human, bank vole or murine PrP carrying mutations associated with these conditions, or other modified forms of mouse PrP [21–34]. The spontaneous development of a transmissible neurodegenerative phenotype has been evidenced in some of these PrP transgenic mouse models while it was either unproven or contested in others. Although instrumental in providing proof-of-principle that genetic human prion disease can be modelled in experimental hosts that express mutated PrP, murine models of these conditions are relatively cumbersome and experimental analysis relatively time consuming. Consequently, a more tractable genetically well-defined animal system is required to search for molecular and cellular pathways of prion-induced neurotoxicity associated with genetic forms of human prion disease.

Increasingly, *Drosophila melanogaster* has been used to model human neurodegenerative disease [35–41]. This has arisen because the brains of *Drosophila* and mammalian species are composed of similar components (i.e. neurons and neuronal circuitry), and the nature of ion channels, neurotransmitters and synaptic proteins are highly conserved between mammals and the fly [42–44]. In addition, *Drosophila* have several important positive experimental advantages including a short lifespan, simple genetics and a well-characterised genome that is amenable to transgenesis [45–47]. We have demonstrated that transmissible mammalian prion disease can be modelled in the fly [48–51]. Our studies have shown that PrP transgenic *Drosophila* develop a neurotoxic phenotype after exposure to exogenous prions that is associated with accumulation of PrP**^Sc^** and is transmissible to PrP transgenic hosts, including mice, two crucial hallmarks of *bona fide* mammalian prion diseases [48–51]. These data show that *Drosophila* possess the cellular and molecular components required for mammalian prion replication. An important unanswered question is whether genetic prion disease, concomitant with the spontaneous formation of transmissible prions, can be modelled in *Drosophila*.

Here, we report the successful generation of PrP transgenic *Drosophila* that provide a novel host system to model genetic human prion disease. We have used pUAST/PhiC31-mediated site-directed mutagenesis to generate *Drosophila* transgenic for murine or hamster PrP that carry single-codon mutations associated with FFI (D178N) or fCJD (E200K) human prion disease. Mouse or hamster PrP harbouring these mutations showed mild Proteinase K (PK) resistance when expressed in the fly. *Drosophila* transgenic for FFI or fCJD variants of mouse 3F4 or hamster PrP exhibited a progressive decline in locomotor ability during adulthood that increased in severity as the flies aged. This severity of effect was more pronounced in *Drosophila* that expressed PrP harbouring the fCJD rather than the FFI mutation, and was more severe in flies that expressed hamster rather than mouse prion protein that harboured these mutations. Significantly, the mutant PrP-mediated neurotoxic fly phenotype was transferable to recipient *Drosophila* that expressed the wild-type form of the transgene. Collectively, these novel data are indicative of the spontaneous formation of a PrP-dependent neurotoxic phenotype in FFI- or fCJD-PrP transgenic *Drosophila* and show that genetic human prion disease can be modelled in this invertebrate host.

## Materials and methods

### PrP amino acid numbering

Species-specific amino acid numbering for mouse, hamster and human PrP is used throughout this study [52].

### Mouse and hamster PrP transgenes

We generated a panel of transgenes that comprised DNA encoding mature mouse or hamster PrP flanked by the coding sequence of an autologous N-terminal leader peptide and a C-terminal GPI (glycosylphosphatidylinositol) signal sequence. The mouse and hamster PrP transgenes carried single-codon mutations that corresponded to the human PrP mutations D178N or E200K, which are associated with inherited FFI and CJD, respectively [9–12]. The mouse PrP also carried two further amino acid substitutions L108M and V111M that created the 3F4 epitope that is already present in wild-type human and hamster PrP [53]. The murine PrP used here is hereafter referred to as mouse 3F4 (or mo3F4) PrP. In addition, we generated transgenes that encoded either murine 3F4 PrP or hamster PrP without mutations associated with inherited human prion disease, hereafter referred to as wild-type mouse 3F4 (or mo3F4**^WT^**) PrP or wild-type hamster (or ha**^WT^**) PrP, respectively. DNA encoding these various transgenes was amplified by PCR using as substrate pBSKSII plasmids that contained inserts that coded for each of these different mouse or hamster prion proteins and oligonucleotide primers complementary to the 5′- and 3′-ends of these sequences. For the amplification of mouse PrP DNA, the primers were:

Forward: 5′-GGC GAA TTC ATG GCG AAC CTT GGC TAC TGG-3′
  Reverse: 5′-GTC CGC TCG AGT CAT CCC ACG ATC AGG AAG ATG-3′

For the amplification of hamster PrP DNA, the primers were:

Forward: 5′-GGC GAA TTC ATG GCG AAC CTT AGC TAC TGG-3′
  Reverse: 5′-GTC CGC TCG AGT CAT CCC ACC ATC AGG AAG ATG-3′

The PCR was carried out in the presence of *Pfu* DNA polymerase (Promega) under reaction conditions that comprised an initial denaturation at 95°C for 10 min followed by 35 cycles of denaturation at 94°C for 30 s, primer annealing at 58°C for 30 s, primer extension at 72°C for 1 min and a final extension of the 785 bp PCR product at 72°C for 7 min (see Supplementary Data S2). The forward and reverse primers contained *EcoR1* and *Xho1* restriction sites, respectively, that allowed directional cloning of the PCR product into the *Drosophila* transgenesis vector pUAST*attB*. The reverse primer contained a stop codon ahead of the *Xho1* restriction site. The various pUAST*attB* DNA constructs were subsequently rescued by transformation in DH5α bacteria. Plasmid DNA was isolated from transformed bacteria by an alkaline lysis method using the Qiagen maxiprep kit and the PrP construct insert verified by DNA sequence analysis.

### *Drosophila* S2 cell culture and transfection

*Drosophila* Schneider 2 (S2) cells were maintained in Schneider's *Drosophila* medium (Invitrogen, Karlsruhe, Germany) containing l-Glutamine and supplemented with 10% foetal calf serum. Cells were cultured at 25°C in atmospheric air. S2 cells were plated 1 day prior to transfection. Transient co-transfections of pUAST*attB*-PrP plasmids and the driver plasmid that expresses GAL4 under the control of the actin5C promoter pWA-GAL4 [54] were carried out using Effectene (Qiagen, Hilden, Germany) according to the manufacturers' instructions. S2 cells were analysed at various time points after transfection.

### Immunofluorescent staining and confocal laser scanning microscopy

*Drosophila* S2 cells were plated on ibidi dishes (Planegg/Martinsried, Germany) or coverslips were fixed with 3% paraformaldehyde solution (pH 7.3) at 21°C for 10 min. In some cases, fixed cells were subsequently permeabilised by treatment with 0.1% Triton X-100 at 21°C for 10 min. In cases where antigen retrieval was required, proteins were denatured by the treatment of fixed and permeabilised cells with 6 M guanidinium hydrochloride (GdnHCl) at 21°C for 7 min. Cells were subsequently rinsed with either PBS in the case of non-detergent-treated cells or in the case of detergent-treated samples, with 0.01% Triton X-100 in PBS (PBST). Washed cells were incubated with mouse monoclonal anti-PrP antibody 4H11 [55] and the Golgi-specific rabbit polyclonal antibody GM130 (Abcam, Cambridge, U.K.) at 37°C for 60 min. After three washing steps in PBS or PBST, as appropriate, cells were incubated with Alexa Fluor-488-conjugated goat anti-mouse IgG (Invitrogen, Karlsruhe, Germany) or Cy3-conjugated goat anti-rabbit IgG (Dianova, Hamburg, Germany) at 21°C for 60 min. Nuclei were stained with Hoechst DNA staining dye (Sigma, Taufkirchen, Germany). Confocal laser scanning microscopy was performed on an LSM 700 laser scanning microscope (Zeiss, Göttingen, Germany).

### Generation of PrP transgenic *Drosophila*

Site-specific transformation of pUAST*attB*-PrP constructs into the 51D fly line (y[1] M{vas-int.Dm}ZH-2A w[*]; M{3xP3-RFP.attP}ZH-51D) was performed by Bestgene, Inc. (CA, U.S.A.). Viable lines were maintained as balanced stocks by conventional fly crosses. PCR was performed on genomic DNA from each balanced fly line to confirm the presence of the correct PrP transgene at the 51D site (see Supplementary Information). *Cre*-mediated removal of RFP from the fly genome of each PrP variant and from non-transgenic 51D flies was performed by conventional fly crosses using y[1] w[67c23] P{y[+mDint2] = Crey}1b; sna[Sco]/CyO (Bloomington Stock 766) [48] obtained from the Department of Genetics, Cambridge University, U.K. The *elav-GAL4* fly line (P{w[+mW.hs] = GawB}elav[C155]) was also obtained from the Department of Genetics, Cambridge University, U.K. All fly lines were raised on standard cornmeal media [56] at 20 or 25°C, maintained at low-to-medium density. Flies were used in the assays described below or harvested at various time points and then frozen at −80°C until required.

### Preparation of *Drosophila* head homogenate

Whole flies in an eppendorf tube were frozen in liquid nitrogen for 10 min and then vortexed for 2 min to cause decapitation. Individual fly heads (1 : 1 mixture of male and female) were isolated and placed in clean eppendorf tubes using a fine paint brush. PBS (pH 7.4) was added to give 1 µl/head and homogenates were prepared by manual grinding of fly heads in the eppendorf tubes with sterilised plastic pestles. Homogenates for western blot of PrP were prepared by processing 20 fly heads in 20 µl of lysis buffer [50 mM Tris (pH 7.5), 100 mM NaCl, 0.5% (v/v) Nonidet P-40 and 1 mM 4-(2-aminoethyl) benzenesulfonyl fluoride (AEBSF)] followed by 10 min sonication on ice. Homogenates for protein misfolding cyclic amplification (20 heads prepared in 20 µl of PBS [pH 7.4]) or for fly-to-fly transmission (secondary passage samples; 300 heads prepared in 300 µl of PBS [pH 7.4]) were prepared from *Drosophila* harvested at 30 days of age. In some cases, fly head homogenate was treated with 0–5 µg/ml PK at 37°C for 30 min in lysis buffer without AEBSF prior to western blot analysis. For PCR analysis, five fly heads were homogenised in 25 µl of PK–lysis buffer [10 mM Tris (pH 8), 1 mM EDTA, 25 mM NaCl and 200 µg/ml PK], incubated at 37°C for 30 min and followed by incubation at 80°C for 10 min.

### SDS–PAGE and western blot

Fly head homogenate was mixed with an equal volume of 2×-strength Laemmli loading buffer, boiled for 10 min, cooled on ice and then centrifuged at 13 000×***g*** for 10 min at 18°C to remove debris. Fly head homogenate was subjected to SDS–PAGE run under reducing conditions and western blot as described in detail previously [57], except that the nitrocellulose membranes were probed with a 1 : 2000 dilution of anti-PrP monoclonal antibody Sha31 [58]. For analysis of *Drosophila* S2 cells by western blot, the cells were pelleted, resuspended in SDS sample buffer and sonicated. After boiling, samples were analysed on NuPAGE Novex 4–12% Bis–Tris gels (Invitrogen, Darmstadt, Germany). Proteins were transferred onto a polyvinylidene difluoride membrane (GE Healthcare, Buckinghamshire, U.K.), and PrP was detected by probing with anti-PrP monoclonal antibody 4H11 [55].

### Prion inoculation of *Drosophila*

*Drosophila* at the larval stage of development were exposed to fly head or mouse brain homogenate. In the case of fly-to-fly prion transmission, fly head homogenate contained the equivalent of 20 fly heads (prepared from 30-day-old flies) per 250 µl PBS. In the case of mouse-to-fly prion transmission, homogenate comprised a 10**^−2^** dilution (v/v) in PBS of adult mouse brain. Two hundred and fifty microlitres of either fly head or mouse brain homogenate were added to the top of the cornmeal that contained third instar *Drosophila* larvae in 3″ plastic vials. Flies were transferred to fresh, non-treated vials following eclosion.

### Negative-geotaxis climbing assay

The locomotor ability of flies was assessed in a negative-geotaxis climbing assay [59] that was initiated with 45 (3 × *n* = 15) age-matched, pre-mated female flies in each treatment group. *Drosophila* were placed in adapted plastic 25 ml pipettes that were used as vertical climbing columns and allowed to acclimatise for 30 min prior to assessment of their locomotor ability. Flies were tapped to the bottom of the pipette (using the same number and intensity of taps on each occasion) and then allowed to climb for 45 s. At the end of the climbing period, the number of flies above the 25 ml mark (top), the number below the 2 ml mark (bottom) and the number in between the 2 and 25 ml mark were recorded. This procedure was performed three times at each time point. The performance index (PI) was calculated for each group of 15 flies (average of three trials) using the formula PI = 0.5 × (*n*total + *n*top − *n*bottom)/*n*total where *n*total is the total number of flies, *n*top is the total number of flies at the top and *n*bottom is the total number of flies at the bottom. A PI value of 1 is recorded if all flies climb to the top of the tube, whereas the value is 0 if no flies climb the tube past the 2 ml mark. The mean PI ± SD at individual time points for each treatment group was plotted as a regression line.

### Statistical analysis

Statistical analysis of the data was performed by one-way analysis of variance (ANOVA), together with Tukey honestly significant difference (HSD) for *post hoc* analysis or the paired-samples Student's *t*-test using Prism (GraphPad Software, Inc., San Diego, U.S.A.).

## Results

### Mouse 3F4 and hamster PrP variants

**Figure 1.**
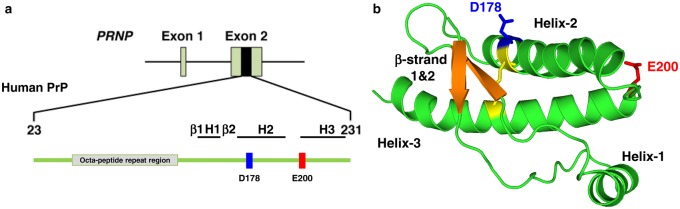
*PRNP* mutations associated with genetic forms of human prion disease. (**a**) Line diagram representation of the human *PRNP* gene and PrP protein, with the amino acid codons in the C-terminal domain of the human prion protein analysed in the present study highlighted. (**b**) Ribbon structure model of the C-terminal domain of human PrP with amino acids D178 (blue) and E200 (red) highlighted. Polypeptide backbone and α-helices shown in green; β-strands are shown in gold, and the disulphide bond between amino acids S179 and S222 shown in yellow.

To model spontaneous prion formation in *Drosophila*, we first generated a panel of DNA constructs that encoded variants of mouse 3F4 or hamster PrP in the fly transgenesis vector pUAST*attB*. These PrP variants contained single amino acid substitutions associated with the human prion diseases FFI and fCJD [9–12]. Figure 1 shows the C-terminal domain location of the human prion protein mutations analysed here. All of the murine 3F4 prion protein variants were generated in mouse PrP that carried two additional amino acid sequence substitutions, namely L108M and V111M. The addition of these two methionine residues in murine PrP created the 3F4 epitope [53], naturally present in human and hamster prion protein, and this protein is hereafter referred to as mouse 3F4 PrP (or mo3F4), where appropriate. The constructs that encoded variants of mouse 3F4 and hamster PrP were generated with DNA encoding their autologous N-terminal leader peptide and C-terminal GPI-signal sequence in order to permit cell-surface protein expression. Mouse PrP (and the 3F4 variant used here) has one less amino acid compared with human and hamster PrP in the N-terminal region of the polypeptide; therefore, species-specific amino acid numbering is used throughout this text [52].

### Mouse 3F4 and hamster PrP transgenes are expressed in *Drosophila* cells

We first verified that the variants of mouse 3F4 and hamster PrP investigated here could be expressed from the pUAST*attB* transgenesis vector. To do so, we transiently transfected *Drosophila* S2 cells with each of the PrP constructs together with the plasmid pWA-GAL4 [54], which was used to drive prion protein expression. We subsequently determined the topological location and relative expression level of each of the different PrP variants by confocal microscopy and western blot analysis, respectively.

**Figure 2.**
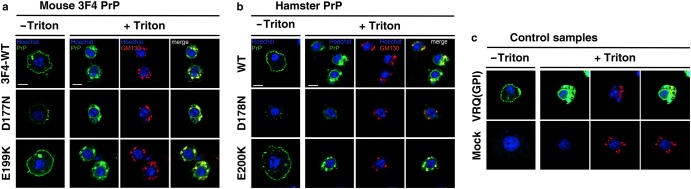
Confocal microscopy of *Drosophila* S2 cells transiently transfected with mouse 3F4 or hamster PrP constructs. *Drosophila* S2 cells were transiently co-transfected with pUAST*attB* plasmids which contained insert DNA that encoded (**a**) mouse 3F4 PrP variants; (**b**) hamster PrP variants or (**c**) ovine VRQ(GPI), together with the pWA-GAL4-driver plasmid. Cells 24 h post-transfection were fixed with or without prior permeabilisation by treatment with Triton X-100. Mock-transfected cells were treated with the Effectene transfection reagent. Prepared cells were subsequently reacted with the anti-PrP monoclonal antibody 4H11, and additionally with the anti-Golgi polyclonal antibody GM130 in the case of permeabilised cells, prior to confocal microscopy. Nuclei were counterstained with Hoechst. Scale bar: 2 μm.

The data in Figure 2 show that variants of mouse 3F4 and hamster (ha) PrP, expressed with their autologous N-terminal leader peptides, were located on the surface of transfected S2 cells, in a manner similar to that of the control PrP expressed with a fly leader peptide [(VRQ(GPI)] [48]. The mo3F4**^CJD^** (E199K) and ha**^CJD^** (E200K) variants were present on the cell surface at a similar level to their respective control proteins mo3F4**^WT^** (wild-type mouse 3F4) and ha**^WT^** (wild-type hamster) PrP. In contrast, the mo3F4**^FFI^** (D177N) and ha**^FFI^** (D178N) were weakly represented at the cell surface of transfected S2 cells. We subsequently investigated whether the reduced cell-surface expression of some of the variants of murine 3F4 and hamster PrP was a consequence of their failure to reach the secretory pathway. To do so, transiently transfected S2 cells were permeabilised and co-stained for the detection of PrP expression and the Golgi marker GM130. The data in Figure 2 show that the mo3F4**^FFI^** and ha**^FFI^** variants showed partial co-localisation with the Golgi.

**Figure 3.**
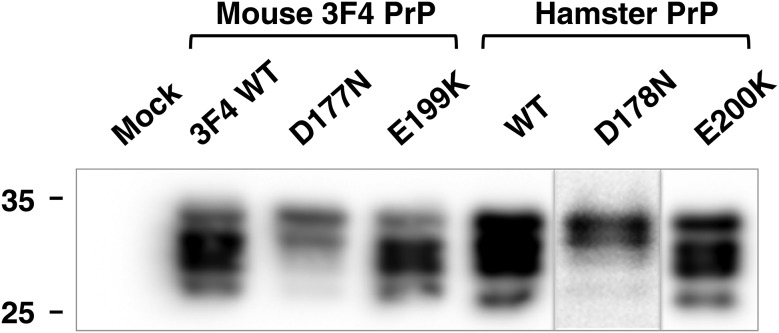
Western blot detection of mouse 3F4 and hamster PrP expression in transiently transfected *Drosophila* S2 cells. *Drosophila* S2 cells were transiently co-transfected with pUAST*attB* that contained insert DNA that encoded mouse 3F4 or hamster PrP constructs, together with the pWA-GAL4-driver plasmid. Control cells were treated with the Effectene transfection reagent. Cell lysates were prepared 24 h post-transfection and subjected to SDS–PAGE, and western blot analysis with the anti-PrP monoclonal antibody 4H11. (The ha**^FFI^** D178N sample track is from a separate gel.) Molecular mass marker values are shown on the left in kDa.

The reduced expression of some of the mouse 3F4 and hamster PrP variants may have arisen because the mutations they carried adversely affected stability of the prion protein [15–19]. Alternatively, the conformation of the PrP variant may have restricted its detection by confocal immunofluorescence. To investigate this, lysates of transfected S2 cells were prepared, denatured and subjected to SDS–PAGE and western blot for the detection of total PrP expression. The data in Figure 3 show that the mo3F4**^CJD^** and ha**^CJD^** variants were present at similar levels in S2 cells compared with their respective control PrP proteins, whereas the mo3F4**^FFI^** and ha**^FFI^** mutants were expressed at a lower level. The data also show that the mo3F4**^FFI^** and ha**^FFI^** variants displayed a different molecular profile in terms of glycosylation to that of the mo3F4**^CJD^** and ha**^CJD^** variants and their respective control PrP proteins.

Collectively, the data in Figures 2 and 3 showed that murine 3F4 and hamster PrP variants were successfully expressed from the *Drosophila* transgenesis vector pUAST*attB* and support the view that mutations in human PrP appear to affect its stability or its ability to traverse the secretory pathway [60].

We subsequently selected the mo3F4**^FFI^** and ha**^FFI^**, mo3F4**^CJD^** and ha**^CJD^** PrP variants, and their respective control proteins mo3F4**^WT^** and ha**^WT^** PrP for expression in *Drosophila*. These particular PrP variants were chosen for three reasons. First, they were efficiently expressed in *Drosophila* cells *in vitro.* Second, they allowed a direct comparison of the pathogenic potential of the same FFI- and fCJD-associated mutations in a mouse 3F4 PrP and hamster PrP context. Third, their use allowed a comparison between the effect of mouse 3F4 PrP variants expressed in *Drosophila* and the same PrP variants expressed in mice where they have been shown to be associated with the spontaneous formation of transmissible prions [27,28].

### Generation of *Drosophila* transgenic for mouse 3F4 or hamster PrP variants

We generated *Drosophila* transgenic for variants of mouse 3F4 or hamster PrP by pUAST*attB*/PhiC31-mediated site-directed mutagenesis [61] whereby the different PrP transgenes were targeted to the same genomic landing site in each respective fly line (see Supplementary Data S1). This transgenesis strategy avoids potential insertional effects, including mutations or alterations in gene expression locally, but also those that affect the expression of the transgenes themselves [62]. We subsequently removed the RFP cassette located at the 51D site by *Cre-*mediated cleavage in each PrP transgenic fly line. This was performed in order to exclude the potential contribution of pigment-associated effects on neuronal integrity and survival [63]. *Drosophila* transgenic for variants of mouse 3F4 or hamster PrP were subsequently crossed with the *elav-GAL4*-driver line to allow pan neuronal expression of these variant prion proteins in the fly.

**Figure 4.**
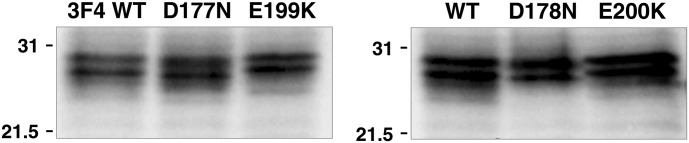
Western blot detection of mouse 3F4 and hamster prion protein expression in PrP transgenic *Drosophila*. Fly head homogenates were prepared from 5-day-old *Drosophila* transgenic for pan neuronal expression of mouse 3F4 PrP (left hand panel) or hamster PrP (right hand panel). Homogenates were analysed by SDS–PAGE and western blot with anti-PrP monoclonal antibody Sha31. The equivalent of six fly heads was run per track. Molecular mass marker values are shown on the left hand side in kDa.

We investigated the molecular profile and expression level of the murine 3F4 and hamster PrP variants in *Drosophila* by immunobiochemical detection methods. The western blot analysis in Figure 4 shows that there was efficient expression of the FFI and fCJD variants of mouse 3F4 and hamster PrP in *Drosophila*, which comprised multiple bands of molecular mass of ∼29–31 kDa, similar to that of mouse and hamster PrP expressed in the fly reported elsewhere [64,65]. The mo3F4**^FFI^** and mo3F4**^CJD^** PrP variants were expressed in *Drosophila* at a level, and with a molecular profile in terms of glycosylation similar to that of mo3F4**^WT^** PrP. Similar trends were seen for the hamster PrP variants ha**^FFI^** and ha**^CJD^** in comparison with ha**^WT^** PrP expressed in *Drosophila*.

Mutations in human PrP that segregate with FFI and fCJD in the natural host are associated with the formation of disease-associated PrP, which shows differences in its state of aggregation and resistance to proteolytic digest compared with the normal form of the prion protein [3]. Consequently, we investigated whether mouse 3F4 or hamster PrP variants expressed in *Drosophila* displayed properties reminiscent of disease-associated prion protein, namely resistance to proteolytic digest.

**Figure 5.**
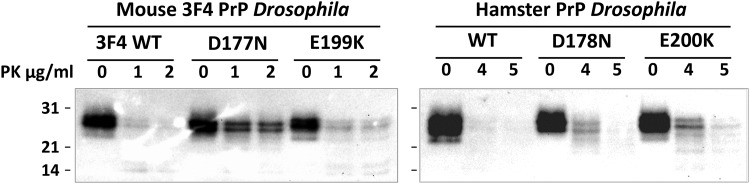
Mildly PK-resistant prion protein in *Drosophila* transgenic for variants of mouse 3F4 or hamster PrP. Head homogenates were prepared from 5-day-old *Drosophila* transgenic for mouse 3F4 or hamster PrP crossed with the *elav*-GAL4 pan neuronal driver fly line and maintained at 25°C. Homogenates were treated with or without PK (0–5 µg/ml) and subsequently analysed by SDS–PAGE and western blot with the anti-PrP monoclonal antibody Sha31. The equivalent of one fly head was run per track. Molecular mass marker values are shown on the left hand side in kDa.

The sensitivity to proteolytic digest of mouse 3F4 or hamster PrP variants expressed in *Drosophila* was examined by PK treatment of fly head homogenate followed by analysis of the reaction products by SDS–PAGE and western blot probed with an anti-PrP monoclonal antibody. The data in Figure 5 show that mouse 3F4 PrP and hamster PrP carrying either an FFI or fCJD-associated mutation displayed an increased resistance to relatively low concentrations of PK that completely digested the respective control PrP protein. The level of PK-resistant prion protein in FFI and fCJD-PrP transgenic *Drosophila* was similar in young adult *Drosophila* (5 days of age, data shown) to that seen in older flies (30 days of age, data not shown). Collectively, these observations showed that variants of mouse 3F4 and hamster PrP were successfully expressed in *Drosophila*, and that PrP mutations associated with genetic prion disease affect the molecular properties of the PrP when it is expressed in the fly.

### Reduced locomotor ability of *Drosophila* transgenic for mouse 3F4 or hamster PrP variants

We next investigated the locomotor ability of *Drosophila* transgenic for variants of murine 3F4 or hamster PrP in order to determine whether FFI and fCJD-associated prion protein mutations induced a spontaneous neurotoxic phenotype in the fly. To do so, we performed a negative-geotaxis climbing assay [50] using adult PrP transgenic *Drosophila*. We compared the response of flies maintained and assessed at 20 and 25°C in an attempt to regulate the expression level of PrP in *Drosophila* through the temperature dependence of GAL4 activity in the fly [66].

**Figure 6.**
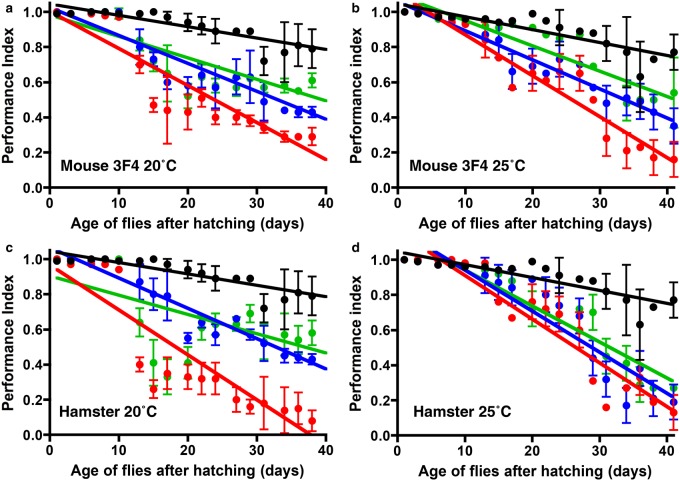
Reduced locomotor ability in *Drosophila* transgenic for variants of mouse 3F4 or hamster PrP. Adult *Drosophila* transgenic for pan neuronal expression of mouse 3F4 PrP (**a**) and (**b**) or hamster PrP (**c**) and (**d**) were maintained at either 20°C (**a**) and (**c**) or 25°C (**b**) and (**d**), and assessed for their locomotor ability by a negative-geotaxis climbing assay. The data shown are linear regression plots of the mean PI ± SD for three groups of flies per time point calculated as described in the Materials and Methods. Green line, mo3F4**^WT^** (**a**) and (**b**) or ha**^WT^** (**c**) and (**d**) PrP flies; red line, mo3F4**^CJD^** or ha**^CJD^** PrP flies (where appropriate); blue line, mo3F4**^FFI^** or ha**^FFI^** PrP flies (where appropriate); black line, non-transgenic 51D flies. Statistical analysis was performed by using the paired-samples *t*-test.

The data in Figure 6 show the locomotor ability, expressed as a PI, of *Drosophila* transgenic for variants of murine 3F4 or hamster PrP as the flies aged after hatching. While expression of mo3F4**^WT^** and ha**^WT^** PrP in *Drosophila* had a negative effect upon the locomotor activity of the fly as reported by others [65,67,68], especially at 25°C, this effect was exacerbated by FFI and fCJD-associated prion protein mutations. Adult *Drosophila* transgenic for mo3F4**^CJD^** PrP showed a significantly enhanced decline in locomotor ability compared with control flies that expressed mo3F4**^WT^** PrP when maintained at 20°C (Figure 6a) and 25°C (Figure 6b; *P* < 0.001 in both cases). Adult flies transgenic for mo3F4**^FFI^** PrP showed a significantly enhanced decline in locomotor ability compared with control flies when maintained at 25°C (Figure 6b; *P* = 0.022). The mo3F4**^CJD^** variant was associated with a more severe neurotoxic fly phenotype compared with the response induced by mo3F4**^FFI^** PrP (Figure 6a,b). Adult *Drosophila* transgenic for ha**^CJD^** PrP showed a significantly enhanced decline in locomotor ability compared with control flies that expressed ha**^WT^** PrP when maintained at 20°C (Figure 6c) and 25°C (Figure 6d; *P* < 0.001 and *P* = 0.0153, respectively). Adult *Drosophila* transgenic for ha**^FFI^** PrP did not show an enhanced decline in locomotor ability compared with control ha**^WT^** PrP transgenic flies when maintained at 20 or 25°C (Figure 6c,d; *P* > 0.05 in both cases). Collectively, these data suggest that expression in *Drosophila* of rodent PrP harbouring mutations associated with genetic forms of human prion disease induces a spontaneous neurotoxic phenotype in the fly. We found no discernible difference in PrP expression level between flies maintained at either 20 or 25°C (data not shown). This suggested that PrP transgenic *Drosophila* maintained at the lower temperature showed less non-specific toxicity, possibly because of reduced metabolic activity compared with flies maintained at the higher temperature.

### Transmission of spontaneously induced neurotoxicity in PrP transgenic *Drosophila*

A hallmark feature of prion diseases is their transmissibility, a characteristic feature mediated by prions including those that develop spontaneously in individuals with genetic forms of these conditions [1,3]. Accordingly, we performed a series of transmission experiments in the fly in order to establish whether the spontaneous neurotoxic phenotype observed in *Drosophila* transgenic for variants of murine 3F4 or hamster PrP was transferable between hosts and therefore potentially prion-mediated.

**Figure 7.**
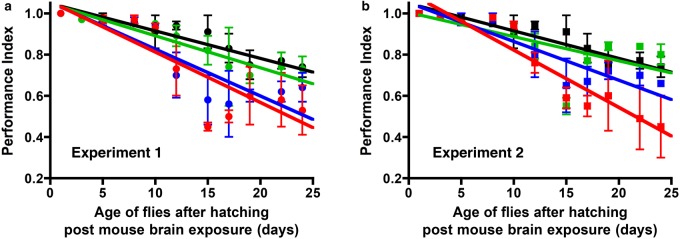
Mouse-to-fly transmission of spontaneous prion-induced neurotoxicity. *Drosophila* transgenic for pan neuronal expression of mo3F4**^WT^** PrP were exposed at the larval stage to mouse brain homogenate from mo3F4**^CJD^** PrP (red line); mo3F4**^FFI^** PrP (blue line); mo3F4**^WT^** (green line) PrP transgenic mice or PrP**^−/−^** (black line) mouse brain homogenate. After hatching, adult *Drosophila* were assessed for their locomotor ability by a negative-geotaxis climbing assay. The data shown are linear regression plots of the mean PI ± SD for three groups of flies per time point calculated as described in the Materials and Methods. Repeat experiments (**a**) 1 and (**b**) 2 are shown, each using different mo3F4 PrP mice brain samples (*n* = 2, all aged ≥350 days). Statistical analysis was performed by using the paired-samples *t*-test.

We first determined whether PrP transgenic *Drosophila* were susceptible to an exogenous source of spontaneously formed prions. To do so, we performed a mouse-to-fly transmission experiment whereby mo3F4**^WT^** PrP transgenic *Drosophila* were exposed, at the larval stage, to brain homogenate from knockin mice that express mo3F4**^FFI^** or mo3F4**^CJD^** PrP variants. Brain homogenates from these mice are an established source of spontaneously generated transmissible prions [27,28]. After hatching, prion-exposed flies were assessed for their locomotor ability by a negative-geotaxis climbing assay. The data in Figure 7 show that adult *Drosophila* transgenic for mo3F4**^WT^** PrP showed a significantly enhanced decline in locomotor ability after exposure at the larval stage to brain homogenate from mo3F4**^FFI^** (*P* = 0.0469 over days 11–25 in Expt. 1 and *P* = 0.0056 over days 1–25 in Expt. 2) or mo3F4**^CJD^** (*P* = 0.0203 over days 1–25 in Expt. 1 and *P* = 0.0097 over days 1–25 in Expt. 2) PrP knockin mice, compared with the response seen after exposure to control brain homogenate from mo3F4**^WT^** PrP knockin mice*.* The response induced by mo3F4**^CJD^** PrP mouse brain material was greater than that induced by similar material from mo3F4**^FFI^** PrP mice, which correlated with the increase in severity of prion disease seen in the original donor mice [27,28]. These data show that PrP transgenic *Drosophila* were susceptible to the neurotoxicity induced by exogenously supplied spontaneously generated prions.

**Figure 8.**
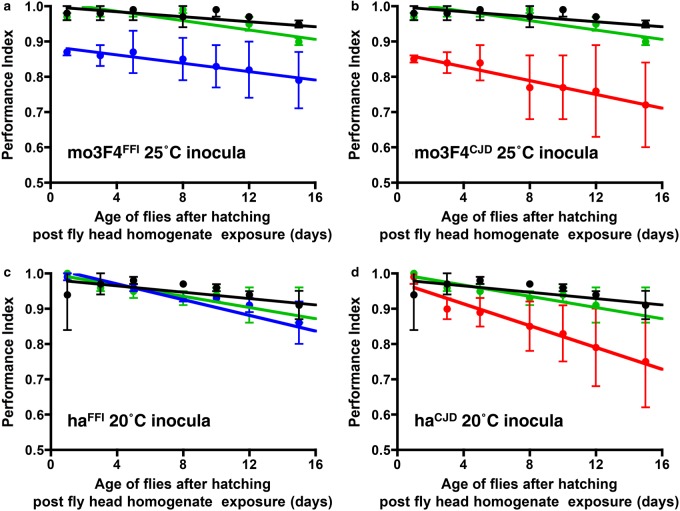
Fly-to-fly transmission of spontaneous neurotoxicity. *Drosophila* transgenic for pan neuronal expression of mo3F4**^WT^** PrP (**a**) and (**b**) or ha**^WT^** PrP (**c**) and (**d**) were exposed at the larval stage to head homogenate from 30-day-old *Drosophila* transgenic for pan neuronal expression of control PrP [mo3F4**^WT^** PrP transgenic *Drosophila* in (**a**) and (**b**), and ha**^WT^** PrP transgenic *Drosophila* in (**c**) and (**d**)], green line; mo3F4**^CJD^** or ha**^CJD^** PrP transgenic *Drosophila* (where appropriate), red line; mo3F4**^FFI^** or ha**^FFI^** PrP transgenic *Drosophila* (where appropriate), blue line. Mouse 3F4 PrP transgenic inocula were from flies maintained at 25°C (**a**) and (**b**); hamster PrP transgenic inocula were from flies maintained at 20°C (**c**) and (**d**). After hatching, adult *Drosophila* were assessed for their locomotor ability by a negative-geotaxis climbing assay. The data shown are linear regression plots of the mean PI ± SD for three groups of flies per time point calculated as described in the Materials and Methods. Black line represents PI of uninoculated non-transgenic control 51D flies crossed with the *elav-GAL4* pan neuronal driver fly line. Statistical analysis was performed by using one-way ANOVA and Tukey HSD for *post hoc* analysis.

We next performed a fly-to-fly transmission study in order to determine whether the spontaneous neurotoxic phenotype seen in *Drosophila* transgenic for murine 3F4 or hamster PrP variants was transferable. In these experiments, *Drosophila* transgenic for either mo3F4**^WT^** or ha**^WT^** PrP were exposed, at the larval stage, to head homogenate prepared from FFI and fCJD mouse 3F4 or hamster PrP transgenic flies, respectively. After hatching, inoculated flies were assessed for their locomotor ability by a negative-geotaxis climbing assay. The data in Figure 8 show that head homogenate from *Drosophila* transgenic for mouse 3F4 PrP variants (Figure 8a,b) and hamster PrP variants (Figure 8c,d) induced a mild but significant accelerated decline in locomotor activity compared with that from age-matched wild-type mouse 3F4 or wild-type hamster PrP flies (*P* ≤ 0.05 in all cases). Head homogenate from flies transgenic for mo3F4**^CJD^** or ha**^CJD^** PrP generally induced a more marked decline in locomotor ability in recipient flies compared with that from flies that expressed mo3F4**^FFI^** or ha**^FFI^** PrP. This correlated with the more pronounced decline in spontaneous locomotor ability exhibited by mo3F4**^CJD^** and ha**^CJD^** PrP transgenic *Drosophila* compared with mo3F4**^FFI^** and ha**^FFI^** PrP transgenic flies (see Figure 6). Furthermore, head homogenate from 30-day-old *Drosophila* transgenic for mouse 3F4 PrP or hamster PrP variants generally induced a more pronounced accelerated decline in locomotor ability in recipient flies compared with the response induced by material from equivalent 5-day-old flies (data not shown). Collectively, these transmission studies suggest that *Drosophila* transgenic for mouse or hamster PrP that harbour either an FFI or fCJD-associated mutation spontaneously generate a transferable toxic moiety, one that accumulates with time as the flies age.

## Discussion

We have generated *Drosophila* transgenic for mouse or hamster PrP that carried single-codon human mutations associated with FFI or fCJD, namely D178N and E200K, respectively. This was performed in order to begin to generate a tractable animal model to probe the mechanism of spontaneous prion-induced neurotoxicity. We used pUAST*attB*/PhiC31-mediated site-specific transgenesis to integrate subtly different PrP transgenes into the same unique landing site in the *Drosophila* genome. This created the first allelic series of fly lines that express PrP from different species harbouring mutations associated with different human prion diseases. Targeted transgenesis enabled a direct comparison of the effects of different PrP variants expressed in *Drosophila*. This approach avoids complications that may arise through random insertional transgenesis, such as silencing, activation or mutation of native genes, or variable transgene expression caused by genomic position effects [62].

The PrP transgenes used here encoded the mature form of mouse 3F4 or hamster prion protein flanked by their autologous N-terminal leader peptide and C-terminal GPI-signal sequences. The rodent PrP transgenes were efficiently expressed in *Drosophila* although the mo3F4**^FFI^** and ha**^FFI^**, but not the mo3F4**^CJD^** and ha**^CJD^**, variants showed a somewhat reduced molecular mass profile to that of mo3F4**^WT^** or ha**^WT^** PrP, respectively. A difference in the molecular profile of mo3F4**^FFI^** PrP and mo3F4**^WT^** PrP was also evident when these proteins were expressed in knockin mice [27]. Confocal microscopy of *Drosophila* S2 cells transiently transfected with the various mouse 3F4 or hamster PrP transgenes showed that these prion protein variants were capable of cell-surface expression. However, while mo3F4**^FFI^** and ha**^FFI^** PrP entered the Golgi apparatus, both were present on the plasma membrane of S2 cells at a lower level compared with their respective control proteins and the mo3F4**^CJD^** and ha**^CJD^** variants. It has been shown that mo3F4**^FFI^** PrP was localised principally in the Golgi and present sparsely at the plasma membrane of mouse granule neurons, whereas the majority of wild-type mouse PrP was present on the plasma membrane and in endosomes, and only a small fraction of the protein was located in the endoplasmic reticulum and Golgi [22]. Furthermore, we have found here that mouse 3F4 PrP and hamster PrP with either an fCJD- or FFI-associated mutation showed a mild resistance to PK compared with the respective wild-type PrP protein. A similar resistance to PK digestion has been shown for mo3F4**^P101L^** PrP expressed in *Drosophila* [67]. These observations are consistent with the view that mutations associated with genetic forms of human prion disease that affect the stability and biochemistry of PrP [15–19,22,24] or its ability to transit the secretory pathway [22,24,60,69] are manifest in the prion protein when expressed in *Drosophila.*

Adult *Drosophila* transgenic for pan neuronal expression of mouse 3F4 or hamster PrP variants displayed a neurotoxic phenotype, evidenced by an accelerated decline in their locomotor ability that increased as the flies aged. Similar defects in locomotor activity have been reported for *Drosophila* transgenic for mouse PrP, with or without a 3F4 epitope, carrying the GSS-associated P102L mutation [67,68,70]. In our studies reported here, the defect in locomotor activity was more evident in *Drosophila* that expressed mo3F4**^CJD^** or ha**^CJD^**compared with those that expressed mo3F4**^FFI^** or ha**^FFI^**. Furthermore, *Drosophila* transgenic for hamster PrP variants generally showed a more severe neurotoxic phenotype than flies transgenic for mo3F4**^FFI^** or mo3F4**^CJD^** PrP. The neurotoxicity we observed in mutant PrP transgenic *Drosophila* is similar to that seen in ovine PrP transgenic *Drosophila* exposed to sheep scrapie prions [48–51]. We reasoned that the phenotype seen in mutant PrP transgenic *Drosophila* was associated with spontaneous prion formation since these flies were not exposed to a source of exogenous prion infectivity. Furthermore, prion-mediated toxicity in hosts that express PrP is considered to be due to conversion of the protein into an abnormal disease-associated conformer concomitant with transmissible prion formation [2,3,5–8]. In this context, we investigated transmissibility of the spontaneous neurotoxic phenotype in PrP transgenic *Drosophila* by secondary passage in recipient PrP transgenic *Drosophila* (i.e. fly-to-fly transmission). Head homogenate from aged adult *Drosophila* transgenic for FFI or fCJD mouse 3F4 or hamster PrP stimulated an accelerated decline in the locomotor ability in recipient *Drosophila* transgenic for mo3F4**^WT^** PrP or ha**^WT^** PrP, respectively, compared with the response seen with control fly head homogenate. We have previously shown that *Drosophila* transgenic for anchorless (**Δ**GPI) ovine PrP develop a spontaneous neurotoxic phenotype that is transmissible to flies that express wild-type ovine PrP [48]. Non-sense mutations in the C-terminal GPI-signal sequence of human PrP, such as Y226X and Q227X transitions, lead to the expression of an anchorless form of the prion protein that are associated with GSS disease [71]. Collectively, therefore, our data presented here, together with our previous findings [48], show that *Drosophila* transgenic for PrP harbouring fCJD- or FFI-associated mutations, or a GSS-related truncated form of PrP, develop a spontaneous transferable neurotoxic phenotype. These observations are compatible with spontaneous prion formation in these novel PrP transgenic hosts.

Mice have been used extensively as an experimental system in attempts to model human genetic prion disease. Accordingly, a large number of PrP transgenic mouse lines have been produced, either by random transgenesis on a PrP**^−/−^** background or direct knockin replacement of the endogenous mouse PrP gene, with a specific prion protein transgene of interest, that harbours mutations associated with genetic forms of human prion disease, or that carry alterations in the PrP polypeptide backbone. However, the majority of these PrP transgenic mouse lines produced little, if any, *bona fide* PK-resistant PrP**^Sc^** [34] and while several of these mouse lines developed neurodegenerative disease phenotypes, only a limited number have successfully been used to demonstrate the generation of infectious prions *de novo*. In one example, knockin mice transgenic for mo3F4**^FFI^** or mo3F4**^CJD^** PrP developed a spontaneous neurotoxic phenotype that was transmissible to mice that express mo3F4**^WT^** PrP [27,28]. In our studies reported here, we have shown that inocula prepared from the brains of knockin mice transgenic for mo3F4**^FFI^** or mo3F4**^CJD^** PrP can induce a neurotoxic phenotype in *Drosophila* transgenic for mo3F4**^WT^** PrP. Furthermore, the time taken to detect spontaneously formed prions by mouse-to-fly transmission, which was completed in weeks, was considerably more rapid than mouse-to-mouse transmission, which required more than 1 year [27,28]. Moreover, large numbers of *Drosophila* could be used to enhance experimental design and improve statistical power, in contrast with mouse experiments that have more significant economic and ethical limitations. These observations highlight the utility of PrP transgenic *Drosophila* to act in conjunction with more sentient mammalian hosts in the study of human prion disease.

In our studies presented here, FFI or fCJD mouse 3F4 and hamster PrP variants were expressed pan neuronally in the fly, a situation analogous to prion protein expression in the natural host with a germ-line *PRNP* mutation. As such, the possibility of cell autonomous mechanisms mediating the adverse effects of PrP misfolding can be envisaged to occur. However, cell non-autonomous mechanisms, such as those associated with the transcellular spread of misfolded PrP, may contribute to the pathogenesis of genetic forms of human prion disease, as they must do in acquired cases, and potentially so in sporadic cases, of prion disease. Cell non-autonomous pathogenic mechanisms are increasingly suggested to play a role in the pathogenesis of more common human protein misfolding neurodegenerative diseases, such as Alzheimer's and Parkinson's disease [72]. The process of transcellular spread of misfolded PrP can readily be studied in *Drosophila* through the facile and versatile nature of transgenesis in the fly, which allows simultaneous expression of mutant and wild-type variants of the prion protein in distinct cell populations with the same host. This type of approach has been used to suggest that phagocytic glia cells in *Drosophila* contribute to the mechanisms of both protein aggregation-related neuroprotection and pathogenesis in protein misfolding neurodegenerative disease [73,74]. The application of this novel experimental approach to the study of genetic forms of human prion disease in the fly will complement our model of transmissible mammalian prion disease that we have established in PrP transgenic *Drosophila* [48–51]. These new invertebrate models of mammalian prion disease will provide us with the opportunity to exploit the power of genetics in the fly to identify potential genetic modifiers of prion-induced neurotoxicity that may serve as candidate diagnostic markers or therapeutic targets of human prion disease and prion-like diseases.

## Abbreviations

ANOVA: analysis of variance
CJD: Creutzfeldt–Jakob disease
fCJD: familial Creutzfeldt–Jakob disease
FFI: fatal familial insomnia
GPI: glycosylphosphatidylinositol
GSS: Gerstmann–Sträussler–Scheinker disease
HSD: honestly significant difference
PBST: Triton X-100 in PBS
PI: performance index
PK: Proteinase K
PrP: prion protein
PrP^C^: normal cellular PrP
PrP^Sc^: abnormal disease-specific conformation of PrP
