# Supplementary material for: Genetic human prion disease modelled in PrP transgenic Drosophila
Source: Biochem J. 2017 Sep 20;474(19):3253–67. doi: 10.1042/BCJ20170462 (PMC5606059; doi:10.1042/BCJ20170462)

Supplementary Information: Generation of *Drosophila* transgenic for mouse 3F4 and hamster PrP varaints

Generation of PrP transgenic *Drosophila*

We generated *Drosophila* transgenic for mouse 3F4 or hamster PrP variants by pUAST*attB* / PhiC31-mediated site-directed mutagenesis in the 51D fly line [61]. Supplementary Data S1 shows an overview of the transgenesis strategy we have used here. The PrP transgenes were generated by PCR from existing pBSKSII plasmids that encoded each of the DNA sequences of interest using forward and reverse primers that contained *EcoRI* and *XhoI* restriction sites, respectively. Supplementary Data S2 shows that these PCR reactions generated a product of 785bp, which corresponded to a DNA fragment that encoded mature mouse 3F4 or hamster PrP flanked by the coding sequence of a leader peptide and GPI signal sequence. The PCR products were subjected to restriction digest with *EcoRI* and *XhoI*, ligated into a similarly digested pUAST*attB* plasmid, and verified by DNA sequence analysis. PrP transgenesis was performed by Bestgene (California, USA).

Confirmation of PrP transgenesis in *Drosophila*

Subsequent to PrP transgenesis in the fly, we used PCR together with DNA sequence analysis, in order to confirm insertion of the correct PrP transgene into each of the different fly lines. PCR was carried out using genomic DNA prepared from balanced PrP transgenic *Drosophila* as template and pUAST-specific oligonucleotides as primers. The pUAST-specific primers were complementary to plasmid DNA sequences adjacent to the 5' and 3' end of the PrP transgene. Supplementary Data S3 shows that a PCR product of the expected size 1046bp was amplified from genomic DNA of each of the mouse 3F4 and hamster PrP transgenic fly lines. No equivalent PCR product was observed in the control reaction using genomic DNA from non-transgenic 51D flies as template. The obtained PCR products were subjected to DNA sequence analysis that confirmed the presence of the correct PrP transgene in each fly line (data not shown).

During pUAST / PhiC31-mediated integration, the single *attP* site in the *ZH-attP-51D* fly line genome recombines with the *attB* site in the pUAST-PrP vector forming *attR* and *attL* sites in the generated transgenic flies as shown in Supplementary Data S1 [61]. In order to confirm the integration of the pUAST-PrP transgenesis vector into the fly genome at the 51D landing site we used PCR with the following set of primers: attP-F specific for the *attP* site in the *ZH-attP-51D* fly line; attL-F specific for the hybrid site *attL* in PrP transgenic fly lines; and primer 51D-R complementary to a site at the 3' DNA end of *attL* site. Supplementary Data S4 show that a PCR product of expected size ~670bp was generated with substrate genomic DNA from each of the ovine PrP transgenic fly lines. A predicted product of ~750bp is generated in this PCR with substrate genomic DNA from non-transgenic 51D flies [48]. We subsequently removed the *lox P*-flanked RFP cassette located at the 51D site [61] by *Cre*-mediated cleavage in each PrP transgenic *Drosophila* line through conventional fly crosses [48]. The various mouse 3F4 or hamster PrP transgenic *Drosophila* were subsequently crossed with the *elav-GAL4*-driver line for pan neuronal expression of prion protein in the fly.

Legends

Supplementary Data S1. PhiC31 / pUAST-mediated site-specific transgenesis in *Drosophila*
The pUAST*attB*-PrP plasmid contains an *attB* fragment, the *white*$^{+}$ marker gene and the PrP transgene. PhiC31 integrase mediates recombination between the pUAST *attB* site and the unique *attP* site in the 51D fly genome [61]. This results in the integration of pUAST*attB*-PrP DNA into the fly genome, thereby creating *attR* and *attL* hybrid sites. The *loxP* sites allow the

elimination of the red fluorescence protein (RFP) and *white*$^{+}$ marker genes after PhiC31-mediated transgenesis. PCR primers attP-F, attL-F and 51D were used to confirm the integration of each PrP transgene in the fly genome at the 51D site. PCR primers pUAST-F and pUAST-R were used to amplify each PrP construct from the genome of transgenic flies.

Supplementary Data S2. Generation of mouse 3F4 and hamster PrP transgenes
PrP transgenes of interest that encoded mature mouse 3F4 or hamster PrP flanked by the coding sequence of a leader peptide and GPI signal sequence were generated by PCR from existing pBSKSII plasmids. The resultant PCR products were analysed on a 2% agarose gel. DNA marker ladder shown on left hand side. Substrate DNA for the PCR was from the following plasmids:
Lane 1: mouse 3F4 wild type
Lane 2: mouse 3F4 D177N
Lane 3: mouse 3F4 E199K
Lane 4: No plasmid DNA substrate control
Lane 5: hamster wild type
Lane 6: hamster D178N
Lane 7: hamster E200K

Supplementary Data S3. Confirmation of variant PrP transgene in *Drosophila*
DNA encoding the PrP transgene of interest was successfully amplified with substrate genomic DNA from each of the mouse 3F4 or hamster PrP transgenic fly lines using primers pUAST-F and pUAST-R (shown in Supplementary Data S1). The resultant PCR products were analysed on a 2% agarose gel and subjected to DNA sequence analysis to confirm the presence of the correct PrP construct in each fly line (data not shown). DNA marker ladder shown on left hand side. Substrate DNA for the PCR was from the following fly lines:
Lane 1: mouse 3F4 wild type
Lane 2: mouse 3F4 D177N
Lane 3: mouse 3F4 E199K
Lane 4: non-transgenic 51D fly line control
Lane 5: hamster wild type
Lane 6: hamster D178N
Lane 7: hamster E200K

Supplementary Data S4. Confirmation of PrP transgene integration into the fly genome
PCR with primers attP-F, attL-F and 51D-R successfully amplified a predicted product of 670bp using substrate genomic DNA from each mouse 3F4 or hamster PrP transgenic fly line. The resultant PCR products were analysed on a 2% agarose gel. DNA marker ladder shown on left hand side. Substrate DNA for the PCR was from the following fly lines:
Lane 1: mouse 3F4 wild type
Lane 2: mouse 3F4 D177N
Lane 3: mouse 3F4 E199K
Lane 4: hamster wild type
Lane 5: hamster D178N
Lane 6: hamster E200K

Materials and Methods

Generation of PrP transgenes and *Drosophila* transgenesis
The generation of PrP transgenes and subsequent fly transgenesis was performed as described in the Materials and Methods section of the main manuscript.

Confirmation of site-specific PrP transgene insertion into the 51D *Drosophila* genome

Site-specific insertion of the various mouse 3F4 and hamster PrP transgenes into the *Drosophila* genome was confirmed by PCR-based procedures. Firstly, 51D site-specific primers (attP-F, attL-F and 51D-R) were used to confirm the integration of the PrP transgene at the 51D site on chromosome 2 of the *Drosophila* genome. Secondly, pUAST site-specific primers (pUAST-F and pUAST-R) were used to identify the PrP transgene located in the *Drosophila* genome.

Primer sequences:

pUAST-F	5’-CTGCAACTACTGAAATCTGCC-3’
pUAST-R	5’-GGCATTCCACCACTGCTC-3’
attP-F	5’-CAACCCTCAGCGGATGCCC-3’
attL-F	5’-GGGCGTGCCCTTGAGTTCTCTC-3’
51D-R	5’-ATCATGCAGGCGAGCGGCTTTC-3’

PCR reaction products were analysed by agarose gel electrophoresis with DNA bands visualised under UV light. A DNA reference ladder (Bioline 1Kb Hyperladder) was included on all gels.

References

1. Bischof, J., Maeda, R. K., Hediger, M., Karch, F. and Basler, K. (2007) An optimized transgenesis system for Drosophila using germ-line-specific phiC31 integrases. Proc Natl Acad Sci U S A. 104, 3312-3317

2. Thackray, A. M., Di, Y., Zhang, C., Wolf, H., Pradl, L., Vorberg, I., Andreoletti, O. and Bujdoso, R. (2014) Prion-induced and spontaneous formation of transmissible toxicity in PrP transgenic Drosophila. Biochem J. 463, 31-40

Supplementary Data S1. PhiC31 / pUAST-mediated site-specific transgenesis in *Drosophila*

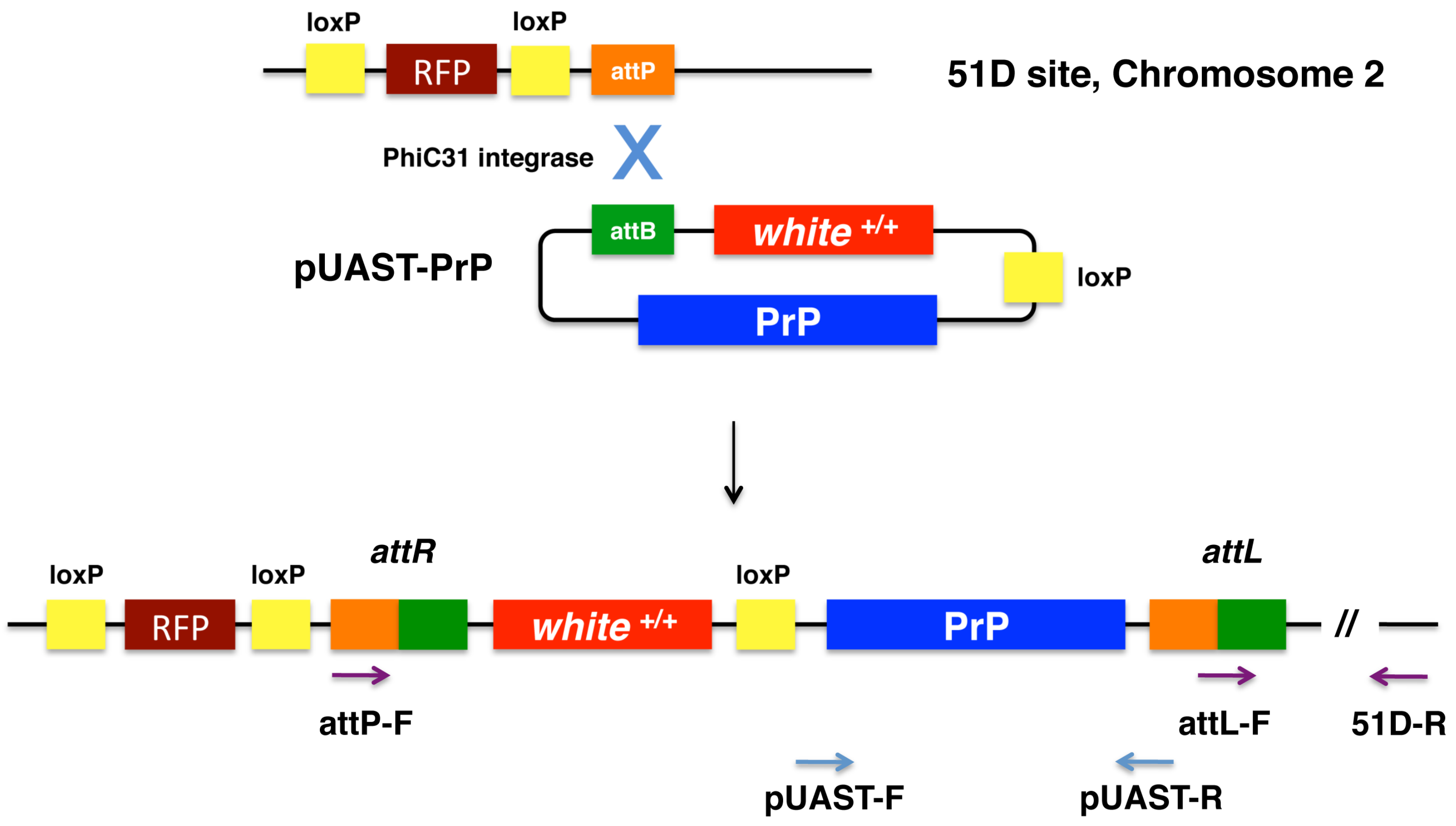

Supplementary Data S2. Generation of mouse 3F4 and hamster PrP transgenes

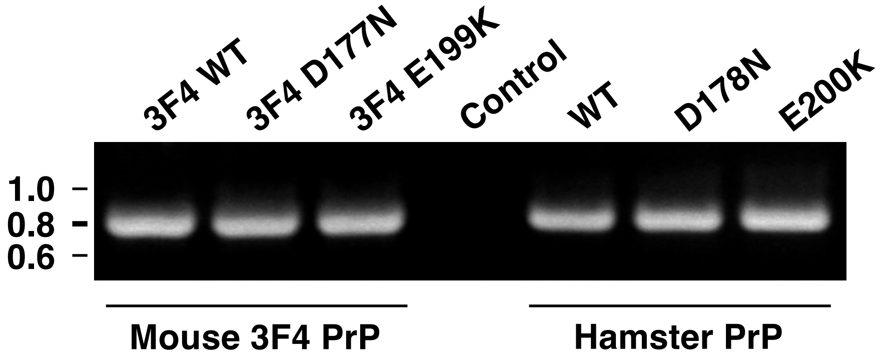

Supplementary Data S3. Confirmation of variant PrP transgene in *Drosophila*

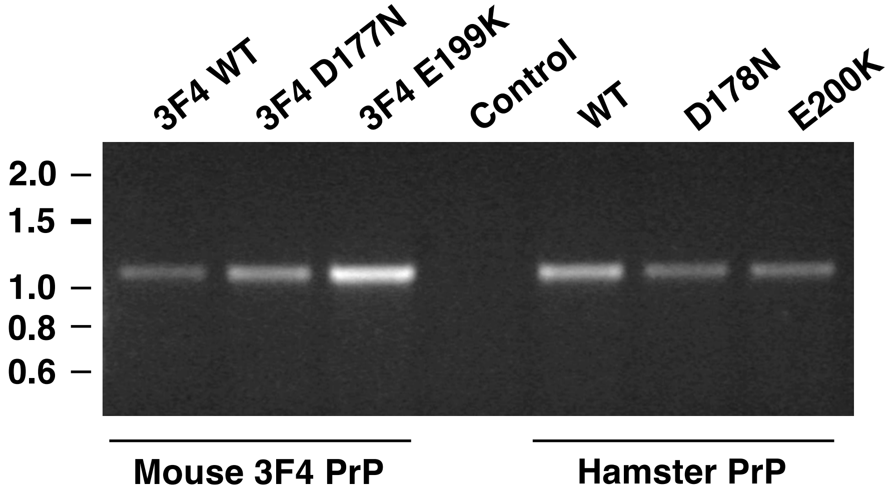

Supplementary Data S4. Confirmation of PrP transgene integration into the fly genome

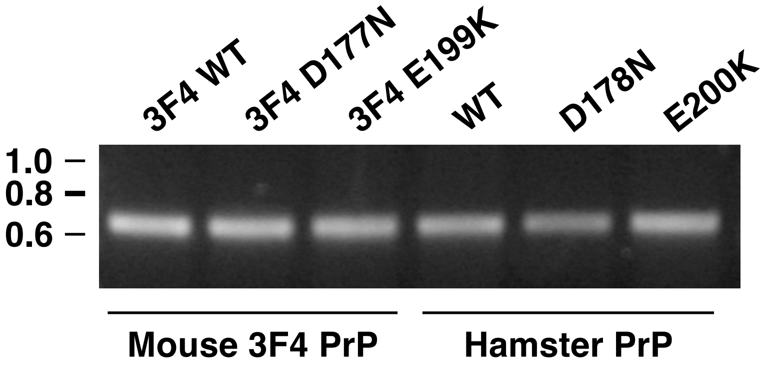

Supplement: Supplementary Information [file BCJ-474-3253-s1.pdf]
